# Concerning Fees

**Published:** 1899-06

**Authors:** 


ARTICLE VI.

### CONCERNING FEES.

BY B.

If we would only stop to compare some of our professional fee customs with the customs of the plumber, we could learn something from the latter. For instance, the plumber's charges for "time" begin the moment he leaves his shop, and saunters to your house. He is "a gentleman of leisure." Every step he takes towards you means a debit in his books, and he is distinguished for the slowness of his steps. Intensely so, he is a strict adherent to the custom of charging for "time," and while he gossips with your cook, or strolls back to his shop for a forgotten tool, his bill runs on all the same. If you examine his bill the "details" amaze you. Every screw, tack, bit of solder, etc., are enumerated. You need not imagine that he will overlook anything or give any of his time, experience or materials for nothing.

But we who are incorporated by law as one of the liberal professions, carry out our liberalism more liberally. Time, experience, and materials are given repeatedly without any request for compensation. Patients treat us with a superciliousness they would not dare show to their plumber. People who expect to be charged the uttermost farthing for every moment of time, and every scrap of material used by the man who is down in their drains, expect the same from the dentist gratis. Indeed it seems to me, as if many dentists act as if they were obliged to apologize to their patients for charging them at all ! "I never charge physicians anything," said a confrere to me. "And does your physician charge you ?" "Oh ! yes," he replied, "but somehow or other I got into the way. Did any one ever hear of such insanity ?

A case came to me several months ago which will illustrate, not only the hereditary mischief done in this way, but the unadulterated gall and ignorance of a certain class of the public. A pompous patient sailed into the office, and dictatorily wanted an appointment to have a tooth filled. "I had it attended to by Dr. —— and he botched it, and I had to get the filling out. Dr. —— then filled it again, and he's another botch. I just want you to fill it again." I explained to her that both of the gentlemen she libelled were among our best and most experienced dentists; and that she doubtless was to blame in not letting them treat the tooth as they probably wanted to treat it. "Oh ! I don't believe in that at all; if a tooth is filled properly it oughtn't to ache." I told her that proper treatment of the roots was necessary; that it would be an expensive experience financially that might or might not succeed, but she replied in her superior way, "All I want you to do is just to fill up the cavity again, and I'll try it." I acceded to her desire under protest, and conditionally, that if my prophetic assurance of trouble was fulfilled, she would at once return. She returned and made an appointment for the purpose of extraction, or beginning the treatment, which ever she would decide upon. She did not come back, but suffered exactly what I predicted, and then placing me and my reputation among her choice list of "botches," she went to another dentist. By that time she had concluded to let the dentist be the judge of the proper way to manage such a tooth, submitted to an expensive treatment and had the tooth saved. This is one of the many cases which provoke us in practice, but which we cannot deal with, simply because we do not get the chance.—*Dominion Dental Journal.*

